# The PanSurg-PREDICT Study: Endocrine Surgery During the COVID-19 Pandemic

**DOI:** 10.1007/s00268-021-06099-z

**Published:** 2021-04-20

**Authors:** K. Van Den Heede, S. Chidambaram, J. Winter Beatty, N. Chander, S. Markar, N. S. Tolley, F. F. Palazzo, J. K. Kinross, A. N. Di Marco, the PanSurg Collaborative and the PREDICT-Endocrine Collaborative, Jasmin Winter Beatty, Swathikan Chidambaram, Sheraz Markar, James K. Kinross, Aimee N. Di Marco, Ayush Kulshreshtha, Rabiya Aseem, Emily K. Deurloo, Nicola C. Quinnen, Nina J. M. DeLa Cruz, Andrew J. Yiu, Natasha Khan, Ola Markiewicz, Ee Teng Goh, Max Denning, Ravi Aggarwal, Sam Mason, Simon Erridge, Simon D. Dryden, Jonathan M. Clarke, Viknesh Sounderajah, Amish Acharya, Simon Rabinowicz, Seema Yalamanchili, Guy Martin, Leigh Warren, Alasdair J. Scott, Elizabeth Burgnon, Sanjay Purkayastha, Robert Mechera, Anthony Glover, Alex Papachristos, Rachel Xuan, Anthony Glover, Bert Dhondt, Mohammed A. Azab, Ahmed Y. Azzam, Dimitris Balalis, Evangelos Fradelos, Dimitris P. Korkolis, Antonia Skotsimara, Efstratia Baili, Eleandros Kyros, Evangelos Felekouras, Ilias Vagios, Lyssandros Karydakis, Maria Mpoura, Athanasios Syllaios, Spyridon Davakis, Theodore Liakakos, Alexandros Charalabopoulos, Andras Fulop, Attila Szijarto, Javeid Bhat, Fazl Parray, Gowhar Aziz, Nisar Chowdri, Rauf Wani, Zameer Shah, Syed Muzamil Andrabi, Asif Mehraj, Amy Fowler, Ali Chaudhary, Ben Murphy, Dayna van der Hoef, Eanna Ryan, Ellen O′Beirn, Fadi Marzouk, Kevin McKevitt, Kulsoom Nizami, Harleen Grewal, Orla Hennessy, Yasmine Roden, Sami A. Elwahab, Christopher Collins, Souad Ayed, Hajer Alwade, Messedah Aldaya, Nasir A. Magboul, Alshahrani Mushabab Ali, Ewout W. Ingwersen, Floor Meijer, Els J. M. Nieveen Van Dijkum, Daan M. Voeten, Suzanne S. Gisbertz, Mark I. Van Berge Henegouwen, Anton F. Engelsman, Ali Yalcinkaya, Can Sahin, Mesut Yavas, Aydin Yavuz, Huseyin Gobut, Hasan Bostanci, Mustafa Sare, Osman Yuksel, Ramazan Kozan, Saygin Altiner, Sezai Leventoglu, Ali Kocataş, Mehmet A. Bozkurt, Yasin Kara, Engin Aybar, Ahmet Can Sari, Elif M. Colak, Siobhan Rooney, Malith Nandasena, Anita Balakrishnan, Mechteld C. De Jong, Radu Mihai, Shahab Khan, Klaas Van Den Heede, Nikita Chander, Swathikan Chidambaram, Fausto Palazzo, Neil Tolley, Aimee Di Marco, Kristie Parkins, Naomi Spencer, Richard J. Egan

**Affiliations:** 1 Department of Endocrine Surgery, Hammersmith Hospital, 72 Du Cane Rd, London, W12 0HS UK; 2 Department of Surgery and Cancer, Imperial College, London, UK

## Abstract

**Background:**

In the midst of the COVID-19 pandemic, patients have continued to present with endocrine (surgical) pathology in an environment depleted of resources. This study investigated how the pandemic affected endocrine surgery practice.

**Methods:**

PanSurg-PREDICT is an international, multicentre, prospective, observational cohort study of emergency and elective surgical patients in secondary/tertiary care during the pandemic. PREDICT-Endocrine collected endocrine-specific data alongside demographics, COVID-19 and outcome data from 11–3-2020 to 13–9-2020.

**Results:**

A total of 380 endocrine surgery patients (19 centres, 12 countries) were analysed (224 thyroidectomies, 116 parathyroidectomies, 40 adrenalectomies). Ninety-seven percent were elective, and 63% needed surgery within 4 weeks. Eight percent were initially deferred but had surgery during the pandemic; less than 1% percent was deferred for more than 6 months. Decision-making was affected by capacity, COVID-19 status or the pandemic in 17%, 5% and 7% of cases. Indication was cancer/worrying lesion in 61% of thyroidectomies and 73% of adrenalectomies and calcium 2.80 mmol/l or greater in 50% of parathyroidectomies. COVID-19 status was unknown at presentation in 92% and remained unknown before surgery in 30%. Two-thirds were asked to self-isolate before surgery. There was one COVID-19-related ICU admission and no mortalities. Consultant-delivered care occurred in a majority (anaesthetist 96%, primary surgeon 76%). Post-operative vocal cord check was reported in only 14% of neck endocrine operations. Both of these observations are likely to reflect modification of practice due to the pandemic.

**Conclusion:**

The COVID-19 pandemic has affected endocrine surgical decision-making, case mix and personnel delivering care. Significant variation was seen in COVID-19 risk mitigation measures. COVID-19-related complications were uncommon. This analysis demonstrates the safety of endocrine surgery during this pandemic.

**Supplementary Information:**

The online version contains supplementary material available at 10.1007/s00268-021-06099-z.

## Introduction

The novel coronavirus disease 2019 (COVID-19) has severely impacted the delivery of safe surgical care. During the pandemic, although there was an overall depletion of resources in hospitals, patients have continued to present with surgical pathology. Consequently, this has resulted in the large-scale disruption of elective surgical services globally [1]. Currently, several studies are being carried out to further understand the impact of the pandemic on the delivery of surgical services at a subspecialty level [2, 3]. The World Health organization (WHO), National Health Service (NHS) in the UK, American College of Surgeons (ACS) and Royal Australasian College of Surgeons (RACS), amongst others, have published specialty-specific guidance on patient prioritization during the pandemic [4–10]. However, the extent of adherence to these guidelines and its impact on patient care and outcomes has not been fully reported.

PanSurg-PREDICT was founded by the PanSurg collaborative to build a novel risk prediction tool to provide surgeons with more accurate estimates regarding the potential risk of complications and mortality in patients, with or without COVID-19 [11]. It collects and analyses world-wide data regarding management and outcomes of patients with surgical pathology, as well as the effects of workforce planning and resource re-allocation in the context of the COVID-19 pandemic. Specifically, in the field of endocrine surgery, there is a paucity of data reporting on the management of patients with endocrine surgical pathology. The aim of this study was to evaluate which patients were selected for endocrine surgery and what precautions were taken to mitigate the risk of COVID-19. Through this, we hope to gain an insight into endocrine surgical practice during the pandemic and establish the extent of COVID-19-related peri-operative morbidity.

## Materials and methods

PanSurg-PREDICT is an international, multicentre, prospective, observational cohort study of emergency and elective surgical patients in secondary and tertiary care during the pandemic. An open invitation to endocrine surgery units was extended via the PanSurg website, social media, to pre-existing PanSurg registrants in other specialties and via national endocrine societies. PREDICT-Endocrine collected data for patients undergoing endocrine surgery over a 6-month period from 21 March 2020 until 13 September 2020. Patient data collection required ethics approval from the local Research and Development (R&D) departments.

### COVID-19 risk assessment

COVID-19 risk was assessed using results of swab tests; imaging results; and self-isolation measures. COVID-19-related morbidity, readmission to hospital or ICU, and clinic follow-up details were also included.

### Data collection

Data collection was performed on the GDPR-compliant platform REDCAP [12, 13]. Data fields included baseline demographic details, details of presentation, management and operative assessment, surgery and outcomes, deferral from surgery, influence on decision to admit and operate, ICU and departmental capacity, prioritizing information regarding emergency admissions, possible cancer diagnosis, multidisciplinary team discussion, TNM classification and NHS priority levels. Endocrine-specific data were incorporated within the overall PanSurg database including indication for surgery, Thy/Bethesda classification, planned procedure, surgical approach, voice changes, vocal cord checks, blood loss, return to theatre, hypocalcaemia, hypothyroidism and pathology reports. Patients were subcategorized into thyroid, parathyroid and adrenal cohorts.

### Statistical analysis

Data were analysed using Microsoft Excel 2019 and Stata MP 14 (Stata Corporation). All quantitative data are presented as median (range). The Chi-square test was used to measure differences between patients with and without COVID-19 mitigation measures with statistical significance at *p* < 0.05.

## Results

Three hundred and eighty patients from 19 different centres in 12 different countries were included (Supplementary Table 1). In total, 224 thyroidectomies, 116 parathyroidectomies and 40 adrenalectomies were analysed.

**Table 1:** Overview of the population characteristics

Population characteristics					
	*Adrenal*	*Parathyroid*	*Thyroid*		*Total*
Age (median, yrs)	50 (18–77)	58 (19–84)	47 (17–90)		51 (17–90)
Gender (F:M ratio)	1.35	2.74	2.50		2.39
BMI (median, kg/m2)	25 (19–41)	27 (16–44)	26 (17–49)		27 (16–49)
Ethnicity (%)					
° White	28 (70%)	71 (61%)	147 (66%)	*p* = *.716*	246 (65%)
° Asian	2 (5%)	13 (11%)	29 (13%)		44 (12%)
° Black	3 (8%)	9 (8%)	12 (5%)		24 (6%)
° Other	7 (17%)	23 (20%)	36 (16%)		66 (17%)
Smoking status (%)					
° Non-smoker	24 (60%)	90 (90%)	151 (77%)	*p* = *.001*	265 (79%)
° Ex-smoker	8 (20%)	4 (4%)	27 (14%)		39 (12%)
° Smoker	8 (20%)	6 (6%)	18 (9%)		32 (9%)
Clinical Frailty Scale (median)	2 (1–5)	2 (1–7)	2 (1–9)		2 (1–9)
ASA (%)					
° I	8 (20%)	29 (25%)	100 (46%)	*p* = *.003*	137 (37%)
° II	25 (62%)	72 (63%)	99 (45%)		196 (52%)
° III	6 (15%)	12 (11%)	20 (11%)		38 (10%)
° IV	1 (3%)	1 (1%)	0		2 (1%)
Pos. Respiratory History	5 (12%)	7 (6%)	30 (14%)		42 (11%)
ECG Changes	0	8 (7%)	8 (4%)		16 (4%)
Presentation (%)					
° Emergency	2 (5%)	5 (4%)	4 (2%)	*p* = *.296*	11 (3%)
° Elective	38 (95%)	111 (96%)	220 (98%)		369 (97%)
Possible Cancer Diagnosis	16 (40%)	4 (3%)	122 (54%)		142 (37%)
MDT Discussion (%)	31 (78%)	15 (13%)	149 (67%)		195 (51%)
Deferred from Surgery (%)	5 (12%)	4 (3%)	22 (10%)		31 (8%)
NHS Priority Scale (%)					
° 1b	0	0	1 (1%)	*p* = *.294*	1 (1%)
° 2	18 (58%)	7 (47%)	97 (66%)		122 (62%)
° 3	13 (42%)	8 (53%)	51 (33%)		72 (37%)
Patients (#)	40	116	224		380

‘#’ means number of patients

### ***Characteristics of patients and operative case mix (Table ******1******)***

The median age of patients was 51 (17–90) years, with a slightly older population undergoing parathyroidectomies (58 (19–84) years). Female to male ratio was 2.39 in the overall population, with a lower ratio for the adrenal patients (1.35). Median BMI was 27 (16–49) kg/m^2^. Most patients were White-Caucasian (65%), Asian (12%) or Afro-Caribbean (6%) (*p* = 0.716). Sixty-two percent were from UK centres, 27.11% Australia and the remainder from Europe (Belgium, Greece, Hungary, The Netherlands, Turkey), India and Middle Eastern countries. There was a higher percentage of smokers undergoing adrenalectomies (20%, *n* = 8) compared to thyroidectomies (9%, *n* = 18) and parathyroidectomies (6%, *n* = 6) (*p* = 0.001). The median ‘Clinical Frailty Score’ (CFS) was 2 (1–5). Fifty-two percent of the patients were ASA grade II. The thyroidectomy cohort had more ASA I patients than the parathyroidectomy and adrenalectomy groups (*p* = 0.003). Eleven percent (*n* = 42) patients had a history of lung or respiratory disease or symptoms.

Eleven patients (3%) presented as an emergency. Most procedures were elective cases, including referrals for possible malignancy (*p* = 0.294). Thirty-seven percent (*n* = 142) underwent surgery for presumed cancerous lesions. This was higher in the thyroid group (54%) than the adrenal (32%) or parathyroid (1,7%) cohorts. Half of parathyroidectomies (*n* = 56) were performed in patients with calcium 2.80 mmol/l or greater. Within the UK, over 60% of the operated patients were high risk and required surgery within 4 weeks (NHS CPG 2). Only 1% (*n* = 1) patient required urgent surgery within 72 h (NHS CPG 1). This was a Graves’ patient with a severe thyroid storm and a history of severe adverse effects from medical treatment.

Thirty-one patients (8.1%) had surgery deferred for 1–6 months, but 7.9% eventually underwent surgery. Only 1 patient was still awaiting his surgery at the end of the inclusion period. The decision for surgery was most affected by department capacity (17%) rather than the pandemic itself (7%) or the patients’ COVID-19 status (5%). Patient admission was influenced by department capacity (8%), the pandemic itself (8%) and the patients’ COVID status (8%). Compared to the same time period in 2019, there was a 42% reduction in surgical procedures during the pandemic.

### ***COVID-19 risk mitigation strategies in endocrine surgery patients (Fig. ******1******)***

**Fig. 1:**
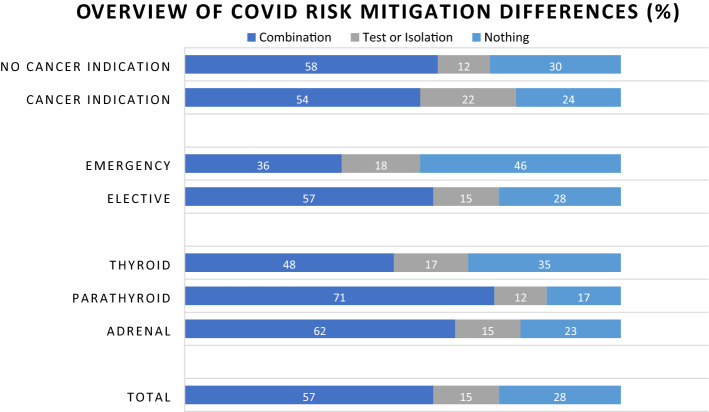
Overview of risk mitigation measure differences

Most patients underwent a nasal/throat swab test (Supplementary Table 2). Fifty-seven percent (*n* = 215) were asked to self-isolate and had at least one COVID-19 test prior to surgery. Fifteen percent (*n* = 59) self-isolated or were tested pre-operatively. Twenty-eight percent (*n* = 106) had an unknown COVID-19 status at time of surgery and were not asked to self-isolate. Only 1 patient underwent surgery with a known positive COVID-19 test prior to surgery. This was again the Graves’ patient with the thyroid storm.

**Table 2:** Comparison between patient cohort with and without COVID-19 risk mitigation measures

Population characteristics measures/no measures				
	Measures	No measures	Chi-square (p values)	Total
Age (median, yrs)	44 (19–85)	54 (17–90)		51 (17–90)
Gender (F:M ratio)	2.22	2.93		2.39
BMI (median, kg/m2)	26 (16–48)	28 (19–49)		27 (16–49)
Ethnicity (%)				
° White	174 (64%)	72 (68%)	*p* = *.019*	246 (65%)
° Asian	27 (10%)	17 (16%)		44 (12%)
° Black	23 (8%)	1 (1%)		24 (6%)
° Other	50 (18%)	16 (15%)		66 (17%)
Smoking status (%)				
° Non-smoker	208 (81%)	57 (73%)	*p* = *.018*	265 (79%)
° Ex-smoker	26 (10%)	13 (17%)		39 (12%)
° Smoker	24 (9%)	8 (10%)		32 (9%)
Clinical Frailty Scale (median)	2 (1–9)			2 (1–9)
ASA (%)				
° I	89 (33%)	48 (46%)	*p* = *.0001*	137 (37%)
° II	158 (59%)	38 (36%)		196 (52%)
° III	20 (7%)	18 (18%)		38 (10%)
° IV	2 (1%)	0		2 (1%)
Pos. Respiratory History	27 (10%)	15 (15%)		42 (11%)
ECG Changes	12 (4%)	4 (4%)		16 (4%)
Presentation (%)				
° Emergency	6 (2%)	5 (5%)	*p* = *.188*	11 (3%)
° Elective	268 (98%)	101 (95%)		369 (97%)
Possible Cancer Diagnosis	108 (39%)	34 (32%)		142 (37%)
MDT Discussion (%)	120 (44%)	75 (71%)		195 (51%)
Deferred from Surgery (%)	6 (2%)	1 (1%)		7 (2%)
NHS Priority Scale (%)				
° 1b	0	1 (1%)	*p* = *.001*	1 (1%)
° 2	86 (72%)	36 (48%)		122 (62%)
° 3	34 (28%)	38 (51%)		72 (37%)
Indication (%)				
° Adrenal	31 (11%)	9 (8%)	*p* = *.003*	40
° Parathyroid	96 (35%)	20 (19%)		116
° Thyroid	147 (54%)	77 (73%)		224
Patients (#)	274	106		380

‘#’ means number of patients

Patients with a possible or confirmed cancer were not more likely to undergo self-isolation and/or to get tested pre-operatively (*p* = 0.185), and this was less likely in emergency presentations than elective cases (*p* = 0.329). However, there was a statistically significant difference in the necessity for self-isolation and/or testing between different surgical procures (*p* = 0.003). Specifically, adherence to risk mitigation measures was less in thyroidectomy patients (*p* = 0.001); White-Caucasians and Asians (*p* = 0.019); ex-smokers (*p* = 0.018); ASA I (*p* = 0.0001); and lower priority guidance grouping (*p* = 0.001). No statistically significant difference was found in emergency presentation between the ‘measures’ and ‘no measures’ group (Table 2).

### Observations of clinical behaviour

The indication for surgery was cancer or a worrying lesion in a majority of thyroid and adrenal cases (Supplementary Table 3) at 61% and 73%, respectively, including phaeochromocytomas. Four patients (10%) had surgery for a confirmed adrenocortical carcinoma. In the thyroid surgery cohort, 137 patients (61%) had surgery for a worrying lesion or confirmed cancer, including completion thyroidectomy for cancer. In this cohort, 116 total thyroidectomies and 101 hemithyroidectomies were included (53% vs 46%). Two Sistrunk’s procedures were performed (1%).

**Table 3:** Pre- and post-operative vocal cord checks in thyroid and parathyroid surgery

Vocal cord checks overall					
	Pre-op		Post-op		
	#	%	#	%	Chi-square
TOTAL	118	35%	44	14%	
Thyroid	94	42%	36	17%	p = .759
Parathyroid	24	21%	8	7%	

Vocal cord checks covid-19 measures/no measures					
	Pre-op		Post-op		
	#	%	#	%	Chi-square
Measures	68	28%	20	10%	p = .037
No Measures	50	52%	5	5%	

Vocal cord checks cancer/no cancer					
	Pre-op		Post-op		
	#	%	#	%	Chi-square
Cancer	53	43%	25	22%	p = .020
No Cancer	63	29%	12	6%	

The indication for parathyroidectomy was primary hyperparathyroidism in 95% (n = 109). Median serum calcium level was 2.80 mmol/l (2.38–3.84 mmol/l). Three percent (*n* = 4) patients had tertiary hyperparathyroidism. Two percent patients (*n* = 2) had surgery for suspected parathyroid cancer. Parathyroid surgery was a bilateral exploration in 63% of cases (*n* = 72) and targeted surgery in the other 37% (*n* = 43). Two patients with malignancy and originally planned for total thyroidectomy, underwent a hemithyroidectomy due to invasion of the recurrent laryngeal nerve (RLN) found at surgery in one and neuropraxia in the other. This decision was taken to avoid the potential for tracheostomy in the unlikely event of a bilateral RLN palsy, specifically because there was concern about how such a patient would fare if they contracted COVID-19.

Consultant-delivered care was the norm during the pandemic (Supplementary Fig. 1). An overwhelming 96% (*n* = 338) procedures were performed under general anaesthesia by a consultant anaesthetist. The primary surgeon was a consultant in 76% of procedures (293 out of 372). Of all procedures, 10% (n = 37) were joint cases with 2 consultant surgeons performing the surgery together.

Pre- and post-operative vocal cord checks in thyroid and parathyroid surgery were evaluated (Table 3). Thirty-five percent (*n* = 118) had a vocal cord check prior to surgery and 14% (*n* = 44) afterwards. There was no statistically significant difference between the thyroid and parathyroid population (*p* = 0.759). A higher percentage of patients had a pre-operative and post-operative vocal cord check in the (possible) cancer group. In the study population with COVID-19 risk mitigation measures, a larger number of post-operative vocal cord checks were performed, in contrast with the higher number of pre-operative vocal cord checks (*p* = 0.037). A follow-up appointment after surgery was planned for 351 patients (98%).

### Morbidity and mortality outcomes

There was no mortality recorded on short-term follow-up (Table 4). Two vocal cord palsies (0.6%) were registered in the thyroid and parathyroid group. After total thyroidectomy, 19% (*n* = 23) patients required calcium substitution. Four percent (*n* = 5) parathyroidectomies (all bilateral explorations) for primary hyperparathyroidism required calcium replacement. The failure to cure rate was 5% for primary hyperparathyroidism. There were 3 neck haematomas post-operatively (0.9%): two that were managed conservatively and one that required surgery for a compressive haematoma post-total thyroidectomy. Five percent (*n* = 19) were admitted to ICU post-operatively (14 planned and 5 unplanned). The one patient that underwent surgery despite a known concomitant diagnosis of COVID-19 went to ICU as he had a severe thyroid storm prior to his surgery. Only 1 unplanned ICU admission occurred due to a COVID-19 infection. One other patient acquired COVID-19 while in hospital. Apart from 2 patients who were transferred to another hospital, all were discharged home (99%). Five patients (1%) were readmitted to the hospital due to surgical morbidity, of which only 1 patient required a readmission for a COVID-19 diagnosis between discharge and the first follow-up appointment.

**Table 4:** Endocrine surgical morbidity during the COVID-19 pandemic

Endocrine surgical morbidity	*Adrenal*		*Parathyroid*		*Thyroid*		*Total*	
	#	%	#	%	#	%	#	%
VC Palsy	/	/	1	1%	1	0.5%	2	0.6%
Hypoparathyroidism	/	/	5	4%	23	19%	28	12%
Failure to cure	/	/	5	5%	/	/	5	5%
Haematoma	0	0%	1	1%	1	0.4%	2	0.5%
Return to theatre	0	0%	0	0%	1	0.4%	1	0.3%
ICU Admission	9	23%	3	3%	8	3%	19	5%
° Planned	8	20%	1	1%	5	2%	14	4%
- COVID-19 related	0	0%	0	0%	1	0%	0	0.5%
° Unplanned	1	3%	2	2%	2	1%	5	1%
- COVID-19 related	0	0%	0	0%	1	0.4%	1	0.5%
Discharged home	40	100%	111	99%	116	99%	267	99%
Hospital readmission	2	5%	1	1%	2	1%	5	1%
° Surgery-related	2	5%	0	0%	2	1%	4	1%
° COVID-19-related	0	0%	1	1%	0	0%	1	0.5%
Hospital-acquired COVID-19 Infection	0	0%	0	0%	1	0.4%	1	0.5%
COVID-19 after discharge	0	0%	1	1%	0	0%	1	0.5%

## Discussion

This international, multicentre, prospective, observational cohort study of emergency and elective endocrine surgical patients is the first to analyse world-wide data regarding management of endocrine surgical patients, risk mitigation measures and the associated outcomes during the COVID-19 pandemic. Over a third of the 380 patients included in the analysis underwent surgery for confirmed or possible cancer. Nearly two-thirds of the patients were classified as requiring surgery within 4 weeks to avoid adverse effects on survival or progression. Eight percent of patients were initially deferred from surgery. According to endocrine surgeons, the pandemic was a considerable factor in the decision to operate in one-third of the patients. Risk mitigation measures differed between countries, with one-third not having to isolate before surgery and nearly one-third having an unknown COVID-19 status at time of surgery. Consultant-delivered care in a majority and post-operative vocal cord checks in a minority are both likely to be sequelae of the pandemic. For example, in the UK around 12% of parathyroidectomy cases and 14% of thyroid cases are performed by a trainee in normal circumstances [14]. The reasons for this shift may be the redeployment of junior staff to ICU or COVID wards or the perception that consultant-delivered care would minimize surgical morbidity. Whatever the reason, the effect on surgical and anaesthetic training is concerning and will need to be monitored. Post-operative vocal cord checks are reported as being performed in 42% in the UK pre-COVID-19 [14], and the authors surmise that the reason for few being performed was their classification as an aerosol-generating procedure (AGP). Despite the preponderance to malignant/suspicious pathology and difference risk mitigation strategies, there were minimal morbidity and no mortality. Thus, endocrine surgery was safely delivered by a hands-on senior clinical approach, despite the disruption and risks caused by the pandemic.

Essential cancer treatments had to continue during the pandemic. Based on national expert opinions, indications for endocrine surgery were updated and recommendations to postpone or prioritize certain procedures were made [4–10]. In the UK, clinical guidelines classified patients requiring surgery into four categories: emergency operations within 24 h; urgent operations within 72 h; elective procedures within 4 weeks; and surgery delayed for 10–12 weeks without adverse outcomes [15–17]. In Australia, the Royal Australian College of Surgeons classified patients into four categories based on clinical urgency, with category 1 requiring surgery before 6 weeks; category 2 before 12 weeks; and category 3 as more than 12 weeks. Category 1 patients were prioritized for surgical intervention during the initial phases of the pandemic [18]. In India, the Association of Surgeons of India categorized patients into three categories based on risk assessment and proposed for surgery to proceed without delay for high-risk category III patients but use a deferred approach for low-risk category I patients for up to 6–8 weeks [19]. A similar approach was also employed in other European countries, including Germany, Italy and Netherlands [20–24]. Hence, the prioritization of patients according to surgical risk and weighing it against the risks of COVID-19 was essential to ensure equitable allocation of surgical care.

From the 8% of patients initially deferred from surgery, most underwent surgery within 3 months. Only one patient had surgery delayed for over 6 months. Reasons for deferral were varied: (a) not deemed sufficiently urgent according to national guidelines; (b) lack of resources (e.g. theatre staff/ICU bed unavailable due to COVID-19); and (c) patient comorbidities make admission/surgery too high risk at time of COVID-19. The decision to offer surgery to a patient was most influenced by department capacity. Hence, surgeons prioritized their patients based on guidelines but were restricted by theatre closures, ICU capacity, ward capacity and/or staff shortages. Once this ‘restriction’ was overcome, the next critical factor for admission was the patients’ COVID-19 status. Our results also show that 60% of cases were not affected by the department capacity or the patient’s COVID-19 status. Thus, resources were available to provide endocrine surgical care for patients who required urgent and semi-urgent surgery.

Several strategies have been implemented to reduce the risk of nosocomial COVID-19 transmission during their inpatient stay with different success rates [25–28]. Sixty percent of patients self-isolated pre-operatively. Seventy percent of patients were tested for COVID-19 in the period before surgery. Thirty percent of patients had an unknown COVID-19 status at time of surgery, and only 7% within this group self-isolated for 14 days. Comparing cohorts with and without risk mitigation measures showed statistically significant healthier patients (ASA I) and less urgent procedures in the group without any mitigation measures. More thyroid surgery was performed in the group without measures, which could be attributed to a younger and healthier cohort. Risk mitigation strategies varied between countries, but not within centres. More post-operative vocal cord checks were performed in the group with known COVID-19 status, while fewer pre-operative vocal cord checks were carried out in the group with risk measures. This probably reflects the ‘fear’ related to a nasal flexible endoscopy during the pandemic and a higher percentage of patients having had their pre-operative vocal cord check prior to the pandemic. Nasal flexible endoscopy is classified as an aerosol generating procedure with risk of viral transmission in some centres [29, 30]. More vocal cord checks were performed in the cancer population (with or without neck dissection), being a known independent risk factor for (temporary) nerve palsy [31]. The lack of vocal cord checks may have influenced the low vocal cord palsy rate after thyroid and parathyroid surgery. The incidence of morbidity was rather small to generate statistically significant comparisons between the two cohorts. Most adrenalectomies were laparoscopic or retroperitoneoscopic, despite concerns of virus particles in surgical smoke [31].

There are some limitations to this study. Firstly, it only includes endocrine surgery units, which tend to be located within a tertiary centre and therefore generates a selection bias, excluding any endocrine surgery which is performed outside of a designated unit. We recognize that while this is a multicentre study, a large proportion of the data is generated from a few countries specifically the UK and Australia: this may be due to the high-volume nature of the units submitting data in these countries or a reflection of the severe impact of COVID-19 on the other countries. This generates a degree of selection bias that limits the generalizability of the data. Furthermore, two countries within the same continent will have variation in the timeline, incidence and management of COVID infections and, most importantly, the broader structure of the healthcare infrastructure, which can influence risk mitigation strategies and patient prioritization. Since we have only reported short-term follow-up data, any influence on cancer outcome, permanent vocal cord palsy and hypoparathyroidism could not be fully addressed. Globally, most centres followed guidance which required a negative COVID test result to qualify for surgery. The testing methodology varied between centres and over the course of the pandemic, leading to varying levels of accuracy and standardization between different centres. This is yet another confounding factor that we could not account for in this study. Future work will focus on reporting the long-term outcomes on morbidity and mortality; the variation in COVID-19 risk mitigation measures; the reduction in surgical services and the impact on surgical training. Prospective data collection will continue to enable comparisons before and after peak incidence during the pandemic.

## Conclusion

The COVID-19 pandemic has a significant impact on surgical management, patient selection and personnel delivering surgical care. Despite a huge variation in COVID-19 risk mitigation measures, COVID-19-related complications were uncommon. This analysis demonstrates the safety of endocrine surgery even during this pandemic.

## Supplementary Information

Below is the link to the electronic supplementary material.

Supplementary file1 (DOCX 157 kb)

## Abbreviations

ASA: American Society of Anesthesiologists
BMI: Body mass index
COVID-19: Coronavirus disease 2019
ECG: Electrocardiogram
FNA: Fine-needle aspiration
GDPR: General Data Protection Regulation
MDT: Multi-disciplinary team
NHS CPG: National Health Service Clinical Priority Grade
NICE CFS: National Institute for Health and Care Excellence Clinical Frailty Score
ICU: Intensive care unit
