# Supplementary material for: The PanSurg-PREDICT Study: Endocrine Surgery During the COVID-19 Pandemic
Source: World J Surg. 2021 Apr 20;45(8):2315–24. doi: 10.1007/s00268-021-06099-z (PMC8057006; doi:10.1007/s00268-021-06099-z)
Supplement: Supplementary file 1 [file 268_2021_6099_MOESM1_ESM.docx]

Supplementary Figure 1. Grade of surgeon and anaesthetist per procedure

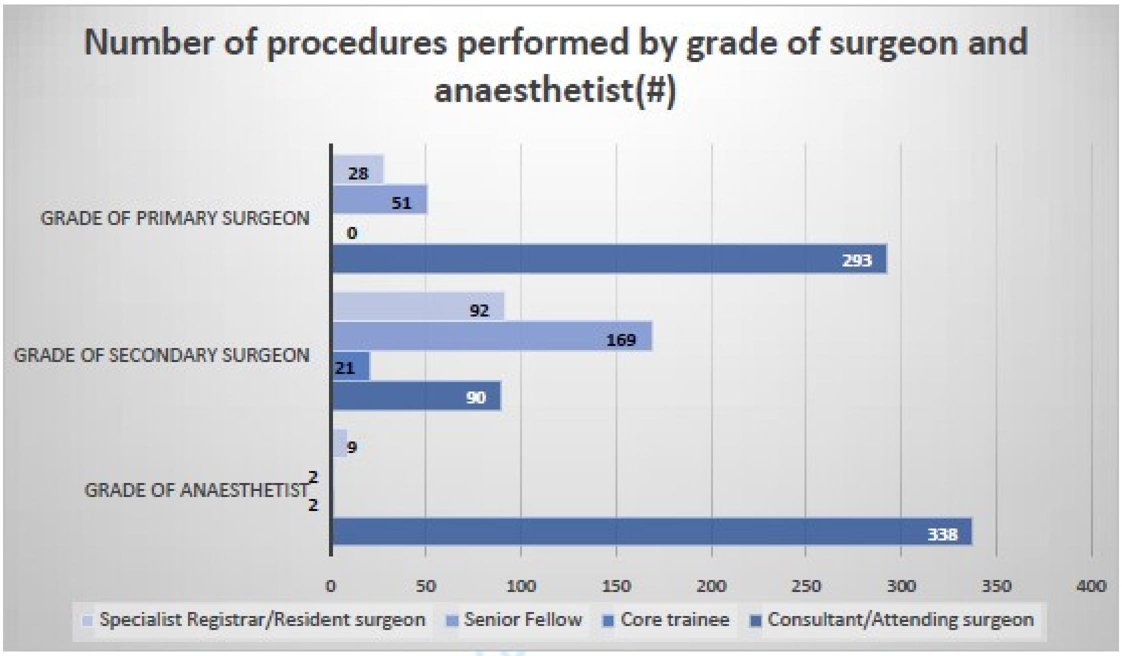

Supplementary Table 1. Overview of the Contributing Centres

CONTRIBUTING CENTRES		
	Surgical Centre	*Country*
**1**	Endocrine Surgery Hornsby Ku-Ring-Gai Hospital	Australia
**2**	Endocrine Surgery Royal North Shore Hospital	Australia
**3**	Surgery AZ Rivierenland, Belgium	Belgium
**4**	Surgical Team, Egypt	Egypt
**5**	General Surgery St Marys, London North West	England
**6**	Endocrine and thyroid surgery, Hammersmith Hospital	England
**7**	HPB Surgery, Addenbrooke's Hospital	England
**8**	Endocrine Surgery Oxford University Hospitals	England
**9**	Surgical Department at "Saint Savvas" Cancer Hospital	Greece
**10**	General Surgery, Laiko General Hospital, University of Athens	Greece
**11**	I st Department of Surgery, Hungary	Hungary
**12**	General surgery, Sher I Kashmir Institute of Medical Sciences, Srinagar	India
**13**	General Surgery UHG	Ireland
**14**	Upper GI Amsterdam UMC	Netherlands
**15**	Aseer central hospital surgery department	Saudi Arabia
**16**	General Surgery, Gazi University, Turkey	Turkey
**17**	General surgery, Turkey	Turkey
**18**	General Surgery STRH, Turkey	Turkey
**19**	General Surgery - Swansea Bay	Wales

For Peer Review

Supplementary Table 2. Overview of COVID-19 Risk Assessment Modalities

COVID-19 RISK ASSESSMENT MODALITIES				
	Swab	*CXR*	*CT*	*Combi*
Presentation	23	1	0	9
Preoperative Asssessment	216	0	0	5
Surgery	40	0	0	2
Discharge	1	0	4	0
**TOTAL**	**280**	**1**	**4**	**16**

Supplementary Table 3. Indications for surgical intervention

INDICATION	#	%
Adrenal		
Adrenocortical Carcinoma	4	10,0
Conn's Adenoma	7	17,5
Control of Cushing's	4	10,0
Metastasis	1	2,5
Phaeochromocytoma	11	27,5
Suspicious on Imaging	13	32,5
Parathyroid		
Renal Hyperparathyroidism	4	3,5
Primary Hyperparathyroidism	109	94,8
Suspicion of Malignancy	2	1,7
Thyroid		
Biopsy Result	86	38,6
Clinically Worrying Lesion	24	10,8
Completion for Cancer	17	7,6
Compressive Symptoms	47	21,1
Quality of Life	19	8,5
Recurrent Cancer	10	4,5
Thyrotoxicosis	20	9,0

For Peer Review
